# ARiRTN: A Novel Learning-Based Estimation Model for Regressing Illumination

**DOI:** 10.3390/s23208558

**Published:** 2023-10-18

**Authors:** Ho-Hyoung Choi, Gi-Seok Kim

**Affiliations:** 1 School of Dentistry, Advanced Dental Device Development Institute, Kyungpook National University, Daegu 41940, Republic of Korea; 2 School of Logos College, Gyeongju University, Gyeongjusi 38065, Republic of Korea; kimgs@gu.ac.kr

**Keywords:** computational color constancy, learning-based estimation model, primary color, appearance, unknown light source, ARiRTN architecture

## Abstract

In computational color constancy, regressing illumination is one of the most common approaches to manifesting the original color appearance of an object in a real-life scene. However, this approach struggles with the challenge of accuracy arising from label vagueness, which is caused by unknown light sources, different reflection characteristics of scene objects, and extrinsic factors such as various types of imaging sensors. This article introduces a novel learning-based estimation model, an aggregate residual-in-residual transformation network (ARiRTN) architecture, by combining the inception model with the residual network and embedding residual networks into a residual network. The proposed model has two parts: the feature-map group and the ARiRTN operator. In the ARiRTN operator, all splits perform transformations simultaneously, and the resulting outputs are concatenated into their respective cardinal groups. Moreover, the proposed architecture is designed to develop multiple homogeneous branches for high cardinality, and an increased size of a set of transformations, which extends the network in width and in length. As a result of experimenting with the four most popular datasets in the field, the proposed architecture makes a compelling case that complexity increases accuracy. In other words, the combination of the two complicated networks, residual and inception networks, helps reduce overfitting, gradient distortion, and vanishing problems, and thereby contributes to improving accuracy. Our experimental results demonstrate this model’s outperformance over its most advanced counterparts in terms of accuracy, as well as the robustness of illuminant invariance and camera invariance.

## 1. Introduction

Colors in a scene image tend to be biased due to unknown light sources, different reflection characteristics of scene objects, and the external spectral sensitivity of diverse imaging sensors. Surprisingly, colors are perceived as constant by the human visual perception system (HVPS) despite unexpected interactions between different light sources. Color constancy is a key attribute of the HVPS that enables consistency in perceiving the original color appearance of an object under any illuminants. This feature has long been drawing much attention from researchers in the computational color constancy community as this characteristic serves as the underlying mechanism for a wide range of computer vision fields and applications. In computer vision, color constancy primarily deals with estimating the illumination color of a scene and reproducing the canonical color of scene objects. In computer vision color constancy, an array of approaches [1,2,3,4,5,6] rely on estimation accuracy to regress the illuminant and use the simple but effective von Kries model [7] to render the scene image. A network is designed to learn regression mapping from the consistent illuminant label or ground truth dataset and thereby perform illumination estimation. To enable networks to perform the most accurate possible estimation, it is also critical to formulate the best possible hypothesis about the illuminant [4]. This is a tough task and requires coping with appearance contradiction and label vagueness. The color appearance of a captured scene object varies significantly depending on the sensitivity of the sensor and illumination spectrum. To reduce such influences, networks are trained on a camera-specific predictor; however, this is deemed ineffective due to the challenge of data demands. Some approaches attempt to make camera-agnostic illuminant predictions and accomplish robust performance. Other approaches, as in ref. [5,6,8,9], have been suggested to address appearance contradiction. In the inception approaches [10,11,12,13], it is worth noting that theoretical complexity is the basis for building highly sophisticated architecture and resultantly improving estimation accuracy. Inception networks have been evolving over time [11,12]. Behind those networks is a split–transform–merge strategy. With a fixed set of receptive field sizes, the network blocks in the architecture perform transformation simultaneously, and the resultant outputs merge in a concatenating manner. They have made progress in estimation accuracy, which is of course attributable to the complexity of the architecture. With the number of receptive fields and their sizes tailored for transformation, the architecture handles data step by step. In this way, constructing more sophisticated architecture has brought incremental progress in accuracy, but not innovation. This begs the question as to the applicability of the network to a new or broader range of tasks or datasets. Inspired to seek the answer and make meaningful enhancements to the color constancy system, a novel learning-based estimation model is introduced, an aggregate residual-in-residual transformation network (ARiRTN) architecture, by combining the inception model with the residual network and embedding residual networks into a residual network. The proposed model has two parts: the feature-map group and the ARiRTN operator. In the ARiRTN operator, all splits perform transformations simultaneously, and the resulting outputs are concatenated into their respective cardinal groups. The proposed architecture is designed to develop multiple homogeneous branches for high cardinality, and an increased size of a set of transformations, which extends the network in width and in length.

This article makes three key contributions, as summarized below.

♦ Creating a novel learning-based estimation model, an aggregate residual-in-residual transformation network (ARiRTN), by combining the inception model with the residual network and embedding residual networks into a residual network.
♦ Experimenting and demonstrating the applicability of the inception model to new tasks and datasets.
♦ Achieving next-level estimation accuracy, as verified by experiments on standard, public datasets, and making a meaningful contribution to the field of computer vision color constancy.

## 2. Previous Works

The Gray-world hypothesis is at the center of traditional color constancy approaches such as GW [14] and its extended versions in ref. [15,16]. These approaches assume that a real-life scene has an achromatic mean of reflection under a neutral source illuminant. The hypothesis uses low-level statistics that describe scene reflectance statistics for achromatic scene color. It is derived from perfect reflectance [17,18] and has been used to develop WP approaches. These approaches feature fast computing speeds and require a small number of free parameters. However, they are too dependent on their hypothesis to cope with unexpected situations outside the conditions of the hypothesis. Some approaches use Bayesian theory in ref. [19] to calculate the posterior distribution for the estimation of the illuminant color and scene surfaces. Bayesian theory was developed to compute the prior distribution of illuminant colors and surface reflectance. The prior distribution is the analytical result of a multivariate truncated normal distribution of the weights of a linear approach. Other approaches [20,21] classify the illuminant color space through the use of the Bayesian framework and train the networks on the histogram frequencies of real-life scenes to generate the surface reflectance prior distributions. For illumination estimation, the approach in ref. [20] uses the prior distribution, which is meant to be a uniform distribution across a subset of illuminant colors, whereas that of ref. [21] uses the empirical distribution of the learning illuminant colors.

In fully supervised works, learning-based approaches [22,23] encompass combinational and direct methods, and their dependence on hand-crafted image features results in performance constraints. Recently, color constancy approaches based on fully supervised convolutional neural networks (CNNs) have made remarkable progress in estimation accuracy. They use either local patches [23,24] or the entire image input [6,25,26,27,28,29,30]. From a color classification perspective, some approaches, including convolutional color constancy [24] and its extended version—the fast Fourier color constancy approach [9]—use a color space on which a histogram shift is used to verify image re-illumination. As a result, they achieve successful and efficient estimation of diverse illumination candidates. The approach in ref. [31] employs a K-means cluster to gather illumination from datasets and adopts a CNN to perform a classification task. Here, the input is a single pre-white balancing image and the output is a K-class probability, and the K-mean cluster predicts each class of illuminants, which accounts for the rendering image.

Finally, the approach in ref. [32] adopts two CNNs for multi-device training: one carries out sensor-independent linear transformation of a 3×3 receptive field size and transforms the RGB color images into a canonical color space, while the other offers the estimated illumination. This approach uses a variety of datasets, except those captured by the test imaging device, and arrives at a successful result. Ref. [33] achieves imaging device invariance by using various samples across diverse imaging devices and datasets in a meta-learning framework. The approach in [34] assumes that standard RGB images gathered from websites are good white-balancing images. They undergo natural de-gamma correction for inverse tone-mapping, and a CNN is used to pick achromatic pixels for illumination estimation. These images were taken using unknown imaging devices and processed with diverse ISP pipelines. Therefore, they might have already been manipulated by unknown software. Nevertheless, this approach makes incremental progress, not innovative. To take estimation accuracy to the next level, this article introduces a novel learning-based estimation model, an aggregate residual-in-residual transformation network (ARiRTN) architecture, by combining the inception model with the residual network and embedding residual networks into a residual network. The proposed model has two parts: the feature-map group and the ARiRTN operator. In the ARiRTN operator, all splits perform transformations simultaneously, and the resulting outputs are concatenated into their respective cardinal groups. The proposed architecture is designed to develop multiple homogeneous branches for high cardinality, an increased size of a set of transformations, which extends the network in width and in length. The next section provides a more detailed elaboration on the proposed approach.

## 3. The Proposed Method

During the last several decades, the inception approach has demonstrated that complexity increases accuracy by carefully designing architectures. Inception networks have evolved over time and their key feature is a split–transform–merge strategy. In their architecture, the network blocks perform transformation simultaneously with a set of specialized receptive fields, and the resulting outputs merge in a concatenating manner. As a result, inception networks bring improved accuracy, which is attributable to their structural complexity. Inspired by the inception network and to take estimation accuracy to the next level, a novel learning-based estimation model is introduced, an aggregated residual-in-residual transformation network (ARiRTN) architecture, by combining the inception model with the residual network and embedding residual networks into a residual network. The proposed model has two parts: the feature-map group and the ARiRTN operator. The subsections that follow discuss the proposed architecture in detail.

### 3.1. Cardinal Groups of ARiRTN

Cardinal groups are formed by separating features into feature-map groups using a cardinality hyper-parameter K as in RexNext [35]. A radix hyper-parameter, R, in this subsection expresses the number of splits within a cardinal group. Hence, the total number of feature-map groups is described as G=KR. Supposing that a cardinal group has a series of transformation $\left\{F_{1},F_{2},\;F_{3},\;\ldots ,\;F_{G}\;\right\}$, a cardinal group is represented as $U_{i}=F_{i}\left(X\right),\;\;for\;i\in \left\{1,\;2,\;3,\;\ldots ,\;G\right\}$. As in ref. [36,37], an integral representation of a cardinal group is obtained through an element-wise summation across all the splits. Suppose that ${\hat{U}}^{k}\in R^{H\times W\times \frac{C}{k}},\;\;for\;k\in 1,\;2,\;3,\;\ldots ,\;K,$ with *H*, *W,* and *C* referring to the output feature-map sizes; the k−th cardinal group is represented as ${\hat{U}}^{k=\overset{Rk}{\underset{j=R\left(k-1\right)+1}{\sum }}U_{j}}$. With channel-wise statistics embedded in the architecture, the global contextual information is obtained through the global average pooling operation in a spatial dimension $s^{k}\in R^{C/K}$. Hence, the c−th constituent is computed as follows [38]:

(1) $$s_{c}^{k}=\frac{1}{H\times W}\sum_{i=1}^{H}\sum_{j=1}^{W}{\hat{U}}_{c}^{K}\left(i,\;j\right)$$

If each feature-map channel is created with a weighted integration across all the splits, the weighted integration of each cardinal group $V^{k}\in R^{H\times W\times C/K}$ is gathered via channel-wise soft attention. Let $a_{i}^{k}\left(c\right)$ represent a soft assignment weight and mapping, $g_{i}^{c}$, decide the weight of each split for the c−th channel based on the global context, $s^{k}$; the c−th channel is described as follows:

(2) $$V_{c}^{k}=\sum_{i=1}^{R}a_{i}^{k}\left(c\right)U_{R\left(k-1\right)+i}$$

where

(3) $$a_{i}^{k}\left(c\right)=\left\{\begin{array}{c} \frac{exp\left(g_{j}^{c}\left(s^{k}\right)\right)}{\sum_{j=1}^{R}exp\left(g_{j}^{c}\left(s^{k}\right)\right)}\;\;\;if\;R>1\;\\ \frac{1}{1+exp\left(g_{j}^{c}\left(s^{k}\right)\right)}\;\;\;if\;R=1\; \end{array}\right.$$

The cardinal groups are then concatenated according to the channel dimension as in $V=concat\left\{V_{1},V_{2},\;V_{3},\;\ldots ,\;V_{K}\;\right\}$. Supposing that the input and output feature maps have the same form, the proposed architecture ultimately generates the output, Y, using skip-connection, described as Y=V+X. Further, a transformation, T, is adopted to modify the output as follows: $Y=T\left(X\right)+V$.

### 3.2. Efficient Implementation of the Proposed ARiRTN Architecture

**Figure 1:**
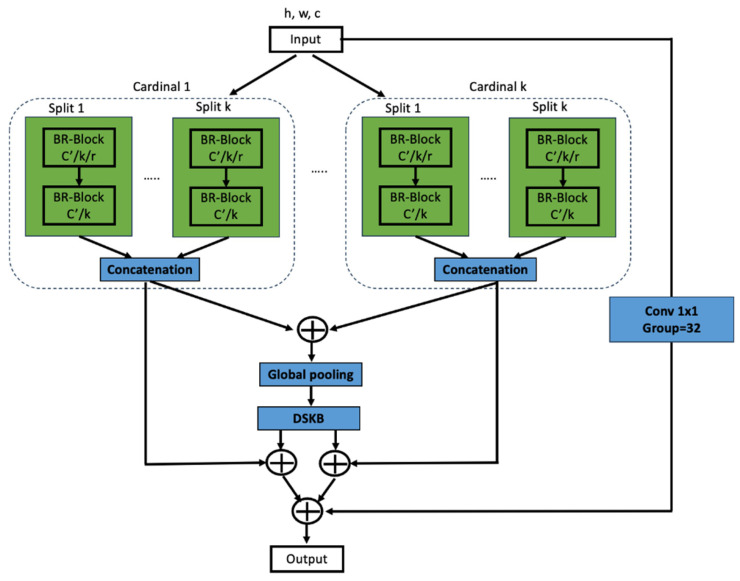
The proposed ARiRTN architecture with radix-major implementation; the feature-map groups, grouped by radix index and cardinality, are sitting next to one another.

What the previous subsection discussed is the layout of cardinality-major implementation. Here, the feature-map groups with the same cardinal index are placed next to one another. Cardinality-major implementation is a simple and intuitive task, but is challenging to modularize and accelerate using CNN architecture. To address this challenge, radix-major implementation is adopted for the proposed architecture. Figure 1 presents the proposed ARiRTN architecture with radix-major implementation. The feature map is separated into several RK groups that have cardinality and radix indexes. The groups with the same radix index are placed next to one another. An add operation is conducted across all the splits. Finally, the feature-map groups are concatenated in a sequence of cardinal numbers; the feature-map groups with identical cardinality indexes merge through the concatenation operation, but not those with different radix indexes. Following the operation of the global pooling layer, K number of successive cardinalities are added up to estimate the attention weights for each split, as shown in Figure 1.

**Figure 2:**
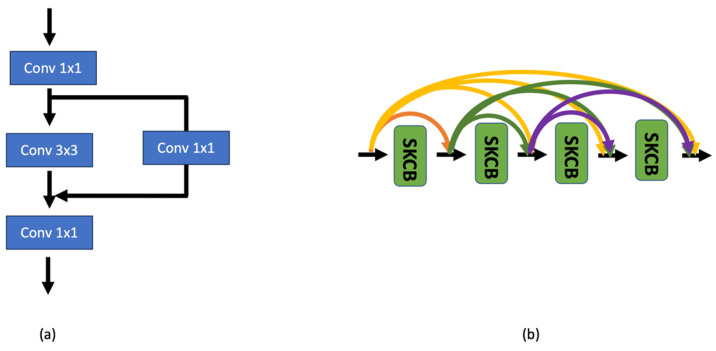
Representation of (**a**) Bottleneck Residual Block (BR-Block) and (**b**) Dense Selective Kernel Block (DSKB) composed of Selective Kernel Convolutional Blocks (SKCBs) from Figure 1.

Figure 2 illustrates the Bottleneck Residual Block (BR-Block) and the Dense Selective Kernel Block (DSKB) shown in Figure 1. The BR-Block is a variant of the residual block that uses a 1 × 1 convolution and is used to create a bottleneck intended to reduce the number of parameters and perform matrix multiplication. The DSKB, which is already introduced in ref. [30], is composed of Selective Kernel Convolutional Blocks (SKCBs). The *l*-th input of the *l*-th SKCB is made up of the feature maps of all its preceding SKCBs, which have undergone the split, fuse, and select. The SKCB has the advantage of adjusting the receptive field size to the changing intensity of the input stimuli. Accordingly, the proposed ARiRTN architecture is expected to obtain stability and robustness in regressing the illuminant. Furthermore, the architecture has potential for broader use in a variety of deep learning applications as it keeps up with the latest network configuration trends.

## 4. Experimental Results and Evaluations

This section discusses the experimental results and evaluations. The proposed ARiRTN architecture experiment was conducted on public, standard datasets of a great number of diverse images taken under a multitude of illumination conditions: the Gehler and Shi illuminant dataset [21] of 0.568 K images that capture a considerable variety of indoor and outdoor scenes, the Gray-ball dataset [39] of 11.340 K illuminant images of diverse scenes, and the Cube+ [40] illuminant dataset of 1365 images that capture different scenes, with their illuminant colors known and additional semantic data used for the purpose of improving the training process towards greater progress in estimation accuracy.

**Figure 3:**
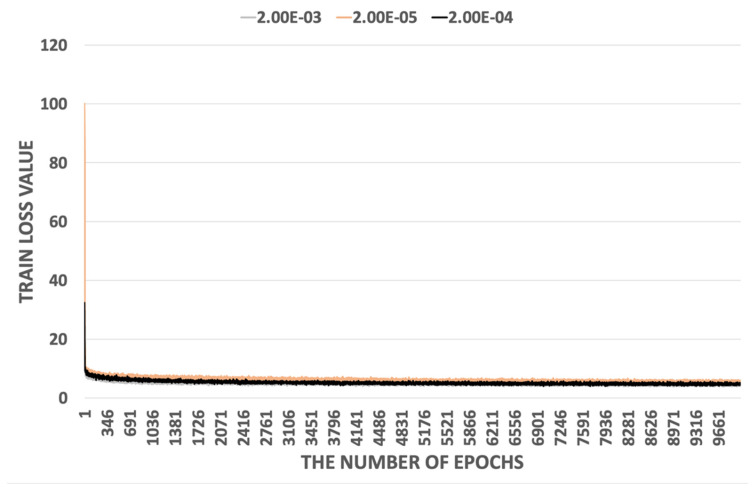
Comparison of initial training rates by calculating their train losses to find one that best fits the proposed architecture.

**Figure 4:**
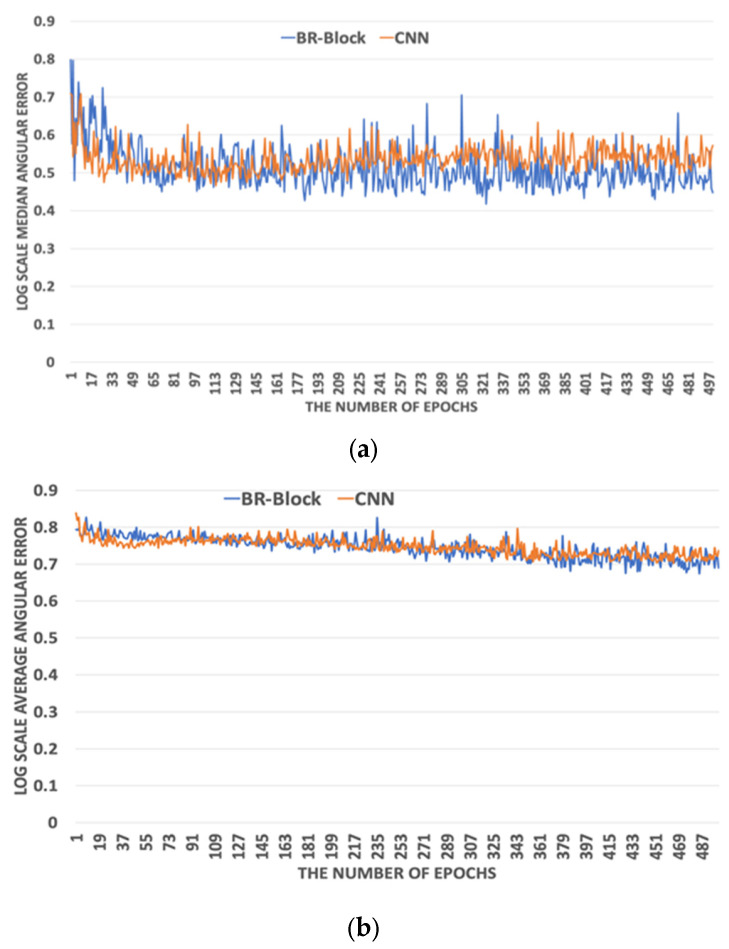
Performance comparison between Bottleneck Residual (BR)-Block and Convolutional Neureal network(CNN) by calculating (**a**) median angular errors and (**b**) average angular errors.

**Figure 5:**
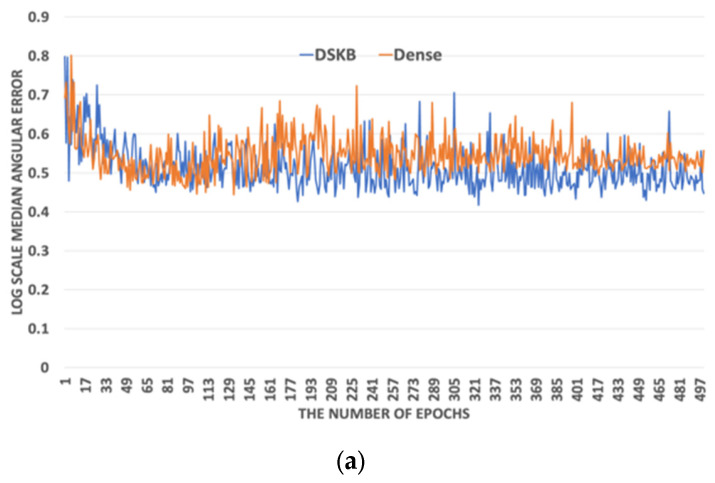

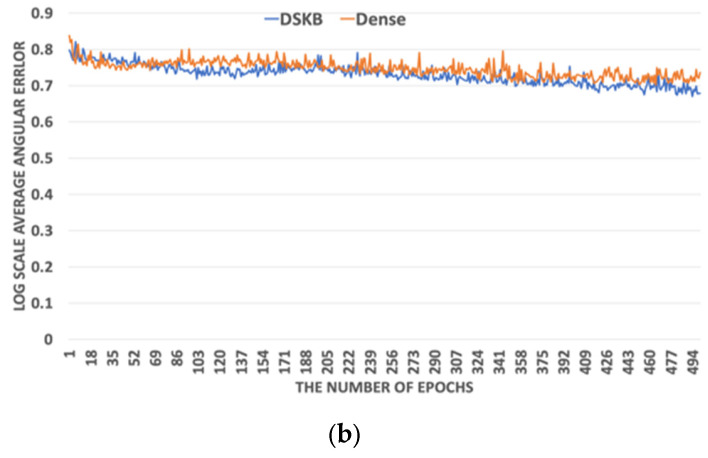
Performance comparison between DSKB and Dense network by calculating (**a**) median angular errors and (**b**) average angular errors.

The proposed ARiRTN architecture runs on the machine learning codes in TensorFlow [41] and is operated in NVIDIA TITAN RTX 24 G. The total training time is 1 day and 11 h with 10 K epochs. In addition to resizing an image into 227 × 227 pixels, the network is set up to have an input batch size of 16. The parameters are optimized through several experiments on the Gehler and Shi illuminant dataset. Figure 3 shows that the proposed ARiRTN architecture tends to converge to zero training loss. Here, with a weight decay of $5\times 10^{5}$ and a momentum of 0.9, several training loss values are compared to determine the optimal initial training rate for the proposed ARiRTN architecture. As highlighted in the previous section, a prominent feature of the proposed architecture is its use of BR-Block and DSKB, as opposed to the CNN and Dense network, their counterparts in conventional network structures, which both consist of 1 × 1 convolutional networks. The proposed ARiRTN architecture employs the BR-Block and DSKB to grow in complexity and increase in width and in depth. As a result, the proposed architecture makes meaningful improvements in estimation accuracy. Figure 4 and Figure 5 depict the comparisons between BR-Block and CNN, and between the DSKB and Dense network, by calculating and representing their median and average angular errors on the logarithmic scale. The Gehler and Shi dataset is used in the comparative experiments of training as well as cross validation, and the median and average angular errors are registered at an interval of 20 epochs.

The next experiments use several standard datasets, the Cube+, Gray-ball, and MultiCam datasets, to compare the performance of the proposed ARiRTN architecture against its most advanced counterparts [42,43,44,45,46,47,48,49,50,51]. In recent decades, the CNN architecture has played an integral role in performing advanced computer vision tasks, including regressing illuminants. However, this approach has struggled with the challenge of accuracy arising from label vagueness caused by unknown source lights, different reflection characteristics of scene objects, and extrinsic factors such as various types of imaging sensors.

Recently, inception approaches have demonstrated that complexity increases accuracy by carefully designing architectures. Inception networks have evolved over time and their key feature is a split–transform–merge strategy. In the architecture, the network blocks perform transformation simultaneously with a set of specialized receptive fields, and the resulting outputs merge in a concatenating manner. As a result, inception networks bring improved accuracy, which is attributable to their structural complexity. Inspired by the inception network and to overcome the limitation of the conventional CNN architecture, a novel learning-based estimation model is introduced, an aggregate residual-in-residual transformation network (ARiRTN) architecture, by combining the inception model with the residual network and embedding residual networks into a residual network. The proposed model has two parts: the feature-map group and the ARiRTN operator.

**Figure 6:**
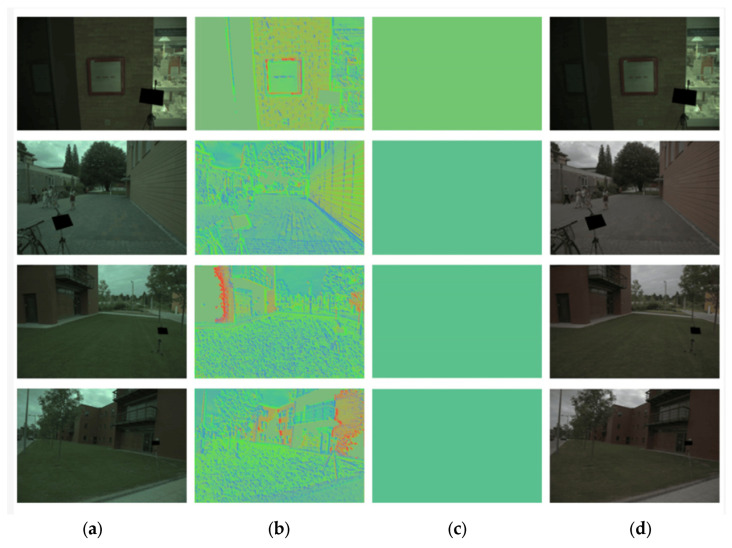
The resulting images at each step delivered by the proposed ARiRTN architecture: (**a**) the original input image (**b**) the estimated illuminant image, (**c**) the ground truth image, and (**d**) the rendered image.

Figure 6 displays the resulting images at each step delivered by the proposed ARiRTN architecture with the Gehler and Shi illuminant dataset. Figure 6 shows (a) the original input image, (b) the resulting image of estimating the illuminant, (c) the ground truth image, and finally, (d) the resulting image after correcting the original image, which ultimately manifests the real-scene image well without an undesired illuminant effect.

Table 1 is a summary table of the comparative analysis between multiple conventional approaches and the proposed ARiRTN architecture in terms of the mean, median, trimean, best 25%, and worst 25%. The experimental results highlight extraordinary performance of the proposed ARiRTN architecture compared to its latest counterparts.

Table 2 is a summary table of the test results that evaluate the proposed ARiRTN architecture against its conventional counterparts, and highlights that the proposed ARiRTN architecture significantly outperforms its conventional counterparts, topping the state-of-the-art approaches in terms of estimation accuracy. Table 1 and Table 2 prove the robust illuminant invariance of the proposed ARiRTN architecture. Table 3 is a summary table of the test results that evaluates the proposed ARiRTN architecture against its conventional counterparts in terms of the angular errors of inter-camera estimation, using a MultiCam dataset that consists of 1365 outdoor images captured using a Cannon 550D camera. The results demonstrate that the proposed ARiRTN architecture surpasses its conventional counterparts in terms of the angular errors of the inter-camera estimation, as well as proving its robustness in terms of illuminant and imaging device invariance.

## 5. Conclusions

In computational color constancy, regressing illumination is a classical approach to manifesting the original color appearance of an object in a real-life scene. However, this approach has struggled with the challenge of accuracy arising from label vagueness, which is caused by unknown source lights, different reflection characteristics of scene objects, and extrinsic factors such as various types of imaging sensors. This article introduces a novel learning-based estimation model, an aggregate residual-in-residual transformation network (ARiRTN) architecture, by combining the inception model with the residual network and embedding residual networks into a residual network. The proposed model has two parts: the feature-map group and the ARiRTN operator. In the ARiRTN operator, all splits perform transformations simultaneously, and the resulting outputs are concatenated into their respective cardinal groups. Moreover, the proposed architecture is designed to develop multiple homogeneous branches for high cardinality, an increased size of a set of transformations, which extends the network in width and in length. Comparative experiments were conducted using the four most popular datasets in the field: Shi’s dataset, the Cube + dataset, the Gray-ball dataset and the MultiCam dataset. The proposed architecture makes a compelling case that complexity increases accuracy by demonstrating outstanding progress in estimation accuracy compared with its previous counterparts. The combination of the two complicated networks, residual and inception networks, is proven to contribute to reducing overfitting, gradient distortion, and vanishing problems, and thereby improving accuracy. These experimental results support this model’s outperformance over its most advanced counterparts in terms of accuracy, as well as the robustness of illuminant invariance and camera invariance. Nevertheless, it is ever meaningful and worthwhile to continue to strive towards creating more advanced learning-based illuminant estimation models and take color constancy to newer heights.

## Figures and Tables

**Table 1:** Comparison of angular errors between multiple conventional approaches and the proposed ARiRTN architecture with Cube+ datasets (lower value means higher accuracy).

Method(s)	Mean	Median	Trimean	Worst 25%	Best 25%
Statistics-Based Approach					
White-Point [42]	9.69	7.48	8.56	20.49	1.72
Gray-World [14]	7.71	4.29	4.98	20.19	1.01
Shade of Gray [15]	2.59	1.73	1.93	6.19	0.46
1st Gray-Edge [16]	2.41	1.52	1.72	5.89	0.45
2nd Gray-Edge [16]	2.50	1.59	1.78	6.08	0.48
Learning-Based Approach					
Fast Fourier CC [9]	1.38	0.74	0.89	3.67	0.19
Sq-FC4 [6]	1.35	0.93	1.01	3.24	0.30
VGG-16 method [43]	1.34	0.83	0.97	3.20	0.28
Multi-Domain LCC [44]	1.24	0.83	0.92	2.91	0.26
One-net [45]	1.21	0.72	0.83	3.05	0.21
Ours	**1.11**	**0.58**	**0.79**	**2.50**	**0.17**

Bold numbers mean the results of the proposed method.

**Table 2:** Comparison of angular errors between the proposed ARiRTN architecture and the conventual approaches with the Gray-ball dataset.

Method(s)	Mean	Median	Trimean	Best 25%	Worst 25%
Support Vector Regression [46]	13.17	11.28	11.83	4.42	25.02
Bayesian approach [21]	6.77	4.70	5.00	-	-
Natural Image Statistics [47]	5.24	3.00	4.35	1.21	11.15
Effective Learning-based [3]	4.42	3.48	3.77	1.01	9.36
CNN-based [25]	4.80	3.70	-	-	-
Ours	**2.85**	**1.53**	**1.63**	**0.42**	**5.95**

Bold numbers mean the results of the proposed method.

**Table 3:** Comparison of inter-camera estimation angular errors between the proposed ARiRTN architecture and the conventional approaches with the MultiCam dataset.

Method(s)	Mean	Median	Trimean	Best 25%	Worst 25%
Gray-World [14]	4.57	3.63	3.85	1.04	9.64
Gamut mapping-based [48]	3.76	2.99	3.10	1.14	7.70
White-Point [51]	3.64	2.84	2.95	1.17	7.48
1st Gray-Edge [16]	3.21	2.51	2.65	0.93	6.61
2nd Gray-Edge [16]	3.12	2.42	2.54	0.86	6.55
Bayesian approach [21]	3.04	2.28	2.40	0.67	6.69
Shade of Gray [15]	2.93	2.24	2.41	0.66	6.31
Spatio-spectral statistics [49]	2.92	2.08	2.17	0.46	6.50
Revisiting gray pixel [50]	2.80	2.00	2.22	0.55	6.25
Quasi-unsupervised [37]	2.39	1.69	1.89	0.48	5.47
CNN-based [25]	1.88	1.47	1.54	0.38	4.90
3-H [51]	1.67	1.20	1.30	0.38	3.78
Fast Fourier CC [9]	1.55	1.22	1.23	0.32	3.66
Sq-FC4 [6]	1.54	1.13	1.20	0.32	3.59
Ours	**1.43**	**1.09**	**1.02**	**0.28**	**3.40**

Bold numbers mean the results of the proposed method.

## Data Availability

http://colorconstancy.com/ (accessed on 2 October 2023).
